# Nanobodies: Next Generation of Cancer Diagnostics and Therapeutics

**DOI:** 10.3389/fonc.2020.01182

**Published:** 2020-07-23

**Authors:** Emily Y. Yang, Khalid Shah

**Affiliations:** ^1^Center for Stem Cell Therapeutics and Imaging, Harvard Medical School, Brigham and Women's Hospital, Boston, MA, United States; ^2^Departments of Neurosurgery, Harvard Medical School, Brigham and Women's Hospital, Boston, MA, United States; ^3^Harvard Stem Cell Institute, Harvard University, Cambridge, MA, United States

**Keywords:** cancer, therapeutics, imaging, immunetherapy, nanobodies

## Abstract

The development of targeted medicine has greatly expanded treatment options and spurred new research avenues in cancer therapeutics, with monoclonal antibodies (mAbs) emerging as a prevalent treatment in recent years. With mixed clinical success, mAbs still hold significant shortcomings, as they possess limited tumor penetration, high manufacturing costs, and the potential to develop therapeutic resistance. However, the recent discovery of “nanobodies,” the smallest-known functional antibody fragment, has demonstrated significant translational potential in preclinical and clinical studies. This review highlights their various applications in cancer and analyzes their trajectory toward their translation into the clinic.

## Introduction

Just under 50 years ago, the “first generation” of therapeutic antibodies consisted of murine-derived, monoclonal antibodies (mAbs), with over 30 mAbs now approved by the Food and Drug Administration (FDA) for clinical use. Despite the clinical potential, their immunogenicity and large size (~150 kDa) became major detriments to their efficacy (1). This prompted the improved “second generation” of utilizing antibody fragments such as the antigen-binding fragment (Fab, ~50kDa) and single-chain variable fragment (scFv, ~30kDa); however, this approach remained limited by a short serum half-life and aggregation-induced immunogenicity (2).

The serendipitous discovery of heavy-chain only antibodies (HcAbs) in camelids sparked the most recent wave of “third generation” antibodies. Compared to conventional mAbs, HcAbs consist of just two heavy chains, with a single variable domain (VHH, ~15kDa) as the antigen-binding region. These nanoscale VHHs were coined the name “nanobodies” and could retain full antigen-binding potential upon isolation, establishing them as the smallest, naturally-derived antigen-binding fragment (3). Nanobodies have spurred the development of commercial companies and have been used in applications such as biosensing, affinity-capture, and protein crystallization; however, their most significant potential lies in therapeutics, especially for cancer. This review highlights how nanobodies have enhanced various cancer diagnostic tools and therapies, both alone, and synergistically. To conclude, an overview of nanobodies in cancer clinical trials is discussed, with an analysis on obstacles, and potential strategies to expedite their implementation as a translational cancer therapy.

## Nanobodies: Types, Structure, and Mechanism of Action

**Figure 1:**
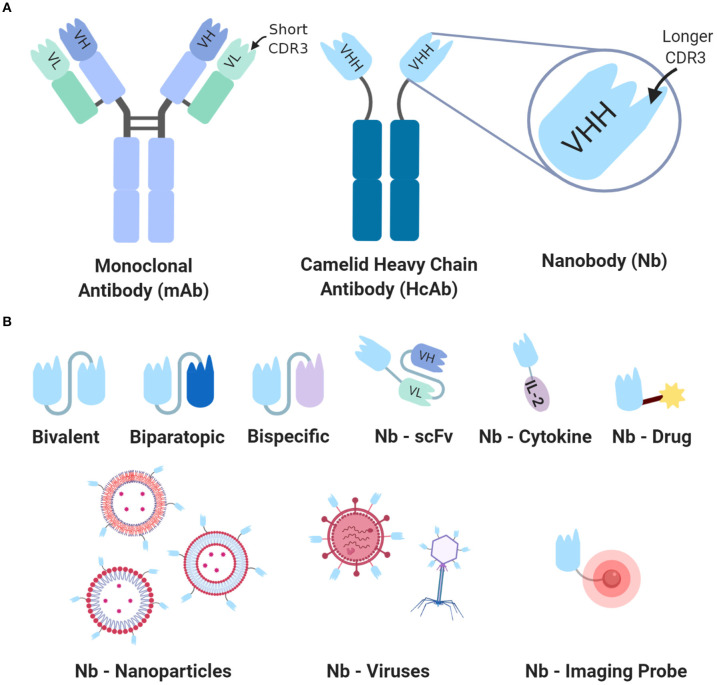
General nanobody structure and types of nanobodies. **(A)** Comparison of the monoclonal antibody (mAb) vs. heavy chain antibody (HcAb) to highlight the structural differences of their respective antigen binding regions. The VHH/Nanobody has a much longer CDR3 loop compared to that of the VH-VL domains in mAbs, providing antigen affinity and access to hidden epitopes. **(B)** A generalized overview of the types of engineered nanobodies to demonstrate how their high modularity enables various modifications. For enhanced antigen avidity, bivalent nanobodies can be created by connecting two identical nanobodies with a linker peptide. Biparatopic nanobodies are a fusion of two nanobodies targeting unique epitopes for the same antigen, with decreased dissociation from the target antigen. Bispecific nanobodies are composed of two nanobodies targeting different antigens and are often utilized as T cell engagers. Nanobodies can also be conjugated to other cancer therapies, nanoparticles, viral vectors, or to imaging agents for targeted tumor visualization. CDR3, complementarity-determining region 3; scFv, short-chain variable fragment; IL-2, Interleukin-2.

Unlike other antibody fragments, nanobodies do not require extensive assembly or molecular optimization to create complex constructs. Possessing such a highly modular nature has propelled a wide array of nanobody-fusion molecules (Figure 1B). Although lacking a VL domain may seem detrimental to antigen binding, nanobodies have evolved to compensate, developing features that also enhance stability, diversity, and binding capacity. In general, antigen specificity is determined at the exposed ends of each variable domain through three peptide loops, or complementarity determining regions (CDRs). The CDR3 loop provides the most significant contribution to an antibody's specificity and diversity, and on average, nanobodies have a much greater CDR3 length compared to that of human VH domains, which strengthens their interactions with target antigens (4) (Figure 1A). Furthermore, their CDR3 regions can form finger-like projections that enable high-affinity binding to traditionally inaccessible cavity-like epitopes (5). Their CDR1 and CDR2 regions also aid in antigen binding, which enables greater paratope diversity than that of mAbs (6).

The inherent properties of nanobodies make them advantageous for cancer applications (Table 1). Their nanoscale dimensions enable deep penetration of tumors, with certain nanobodies able to cross the blood brain barrier (BBB) (7). Nanobodies also retain high affinity and specificity for their target antigens, with low off-target accumulation due to their hydrophilic regions (8). They're also unexpectedly robust due to their high refolding capacity, recovering from chemical denaturation with minimal damage to functionality, although a recent study suggests that thermal denaturation may be irreversible (9). Furthermore, they can tolerate high temperatures (60–80°C, several weeks at 37°C), elevated pressures (500–750 MPa), non-physiological pHs (3.0–9.0), and even the strongest of chemical denaturants (2–3 M guanidinium chloride, 6–8 M urea) (10). From a manufacturing standpoint, nanobodies are simple and inexpensive to produce. Lacking post-translational modifications, nanobodies can be synthesized through microbial systems, with the additional benefit of generating homogeneous products (11).

**Table 1:** Advantages of nanobodies vs. current therapies.

**Clinical application**	**Improvements**
Molecular imaging	• High T/B • Enhanced tumor penetration • Minimal off-target retention • Rapid renal clearance
Intrinsic therapeutics	• Greater paratope diversity • Access hidden epitopes • Can cross BBB • Low off-target accumulation • May overcome mAb-associated resistance
Enhancing immune response	• High degree of modularity • Decreased immunosuppression • Enhanced immune activation • Low off-target accumulation • Synergy with mAbs
Nanobody-secreting SCs	• Enhanced tumor penetration • Synergistic potential of SC-based delivery
Photodynamic therapy	• Enhanced tumor penetration • Rapid renal clearance • Decreased photosensitivity in patients
Drug delivery	• Enhanced drug efficacy • Increased maximum dose tolerance • Improved target specificity • High degree of modularity
Viral vectors	• Enhanced vaccine efficacy • Improved target specificity
Intracellular targeting	• Currently not possible with mAbs • Targets traditionally inaccessible tumor markers • Various delivery options

*T/B, tumor-to-background ratio; BBB, blood brain barrier; mAb, monoclonal antibody; SCs, stem cells*.

## Nanobodies in Cancer Imaging

Much of the focus in cancer is placed on therapeutics, but the diagnostics of tumor imaging are just as critical, as visual knowledge of the tumor's antigen profile is needed to maximize therapeutic efficacy. A variety of imaging modalities are utilized in cancer diagnostics, and molecular imaging techniques have shown potential in improving existing techniques (Table 2). Molecular imaging utilizes a molecular probe that binds to a tumor antigen. Molecular imaging has been extensively explored with mAbs; however, their weak tumor penetration and longer serum half-life are significant obstacles in creating high-contrast images. Thus, nanobodies form quite suitable candidates, ensuring minimal non-target retention to create a high tumor-to-background ratio (T/B) shortly after administration.

**Table 2:** Current cancer imaging techniques.

**Modality**	**Background**
X-Rays	° Based on density-dependent X-ray absorption differences ° Can be used for bone, lung, and breast cancer detection ° Fast, inexpensive, but lower resolution than CT
CT	° 3D reconstruction of X-ray images ° Most commonly used technique for detecting abnormal morphologies, can be combined with PET and SPECT ° Fast, high spatial resolution, inexpensive, but soft-tissue sensitivity is limited by toxicity concerns
PET	° Nuclear imaging agent ° (e.g., ^18^F, ^68^Ga, ^89^Zr) emits positrons ° Superior sensitivity (10^−11^-10^−12^ mol/L) and spatial resolution, but shorter imaging window, expensive
SPECT	° Nuclear imaging agent (e.g., ^99m^Tc) emits gamma rays ° Cheaper than PET, but lacks spatial, and temporal resolution
Optical	° Molecular probes are tagged with fluorescent dyes ° Fast, inexpensive, no radiation, but limited high penetration range (700–900 nm)
MRI	° Utilizes strong magnetic fields ° DW MRI can reliably determine aggression of certain tumors ° Very high spatial resolution, no radiation, but low sensitivity (10^−3^−10^−5^ mol/L), expensive
Ultrasound	° Detects reflected sound waves from tissues ° Mainly used for imaging angiogenesis ° High spatial and temporal resolution, no radiation, portable, inexpensive, but limited to systemic vasculature
Quantum dots^*^	° Fluorescent semiconductor nanocrystals ° Adaptable, superior stability, multiplex detection, but low biocompatibility

*CT, computed tomography; PET, positron-emission-tomography; SPECT, single photon emission computed tomography; nm, nanometer; MRI, magnetic resonance imaging; DW, diffusion weighted*.

* *Still in pre-clinical phase*.

The nuclear techniques of position emission tomography (PET) and single photon emission computed tomography (SPECT) comprise the majority of molecular imaging studies due to their quantitative output, high sensitivity, and clinical relevance. PET proves superior in sensitivity and spatial resolution; however, it's limited by a shorter imaging window and costly implementation. For tracking, nanobodies are tagged with a positron-emitting nuclide (e.g., ^18^F, ^68^Ga, ^89^Zr) for PET, and gamma-emitting nuclides (e.g., ^99m^Tc) are used for SPECT.

Optical imaging, ultrasound, magnetic resonance imaging (MRI), and quantum dots have also been studied with nanobodies. In optical imaging, nanobodies are tagged with fluorescent dyes, and although the technique has weaker penetration, it offers the advantages of flexibility, simplicity, cost-effectiveness, and safety. Ultrasound imaging utilizes reflected sound waves from tissues, and nanobodies have been tagged to contrast agents, microbubbles, and nanobubbles. It is also a comparatively safer technique, but its applications are currently limited to systemic vasculature (12). MRI is a more expensive technique that utilizes strong magnetic fields to generate higher resolution images, and it has been paired with nanobody-coated superparamagnetic nanoparticles (13), magnetoliposomes (14), and fluorescent streptavidin (15) for detecting ovarian tumors. Quantum dots are fluorescent nanocrystals that have recently demonstrated tumor imaging potential for their adaptable properties, superior stability, and multiplex detection; however, their current implementation is limited by their low biocompatibility. Nanobody-conjugated quantum dots targeting carcinoembryonic antigen (CEA) (16), epidermal growth factor receptor vIII (EGFRvIII) (17), and cytotoxic T lymphocyte antigen-4 (CTLA-4) (18) have achieved enhanced targeting with minimal toxicity *in vivo* (17, 18).

### Tumor Identification

Currently, the most advanced of nanobody probes target human epidermal growth factor receptor 2 (HER2) and are in clinical testing. In 2014, a phase I clinical trial tested a ^68^Ga-HER2 nanobody that could detect primary and metastatic tumors without adverse effects (19), leading to a phase II clinical trial (20). Other studies have assessed carbonic anhydrase IX (CAIX) and HER2-CAIX targeting for optical imaging (21). Notably, the HER2-CAIX combination synergistically enhanced the T/B ratio and could also detect lung metastases (22). Additionally, ^89^Zr-HER3 (23), ^18^F-HER2 (24), and ^68^Ga-NOTA-CD20 (25) nanobodies have demonstrated success in various tumor models. Pant et al. (26) developed a novel implementation of anti-EGFR-nanobody-dendritic polyglycerols (dPGs), demonstrating enhanced accumulation *in vivo*. ^99m^Tc-EGFR (27), ^99m^Tc-EGFR-cartilage oligomeric matrix protein (COMP) (28), ^99m^Tc-dipeptidyl-peptidase-like protein 6 (DPP6) (29), ^99m^Tc-mesothelin (30), and ^131^I-HER2 (31) nanobodies nanobody probes have also demonstrated high T/B ratios. Additionally, anti-EGFR nanobody probes have been utilized in dual-isotope SPECT (32) and optical imaging (33), with an enhanced T/B ratio vs. mAb-based probes (32, 33).

### Tumor Stroma Visualization

Tumor treatment resistance is often due to its intimate interactions with the surrounding tumor microenvironment (TME), an amalgam of extracellular matrix (ECM), angiogenesis, and infiltrating immune cells. This TME often accelerates tumor growth while repressing therapeutic efficacy; thus, its visualization is of paramount importance. Imaging tumor angiogenesis has been explored through targeting vascular cell adhesion molecule-1 (VCAM-1), a marker associated with metastasis and immune evasion, and anti-VCAM-1 nanobody-microbubbles have been used for ultrasound imaging of murine carcinomas (12). Nanobody probes targeting immune checkpoints (ICP) CTLA-4 and programmed death ligand 1 (PD-L1) (34–38) have been implemented in nuclear imaging with high T/B ratios (39, 40), and a phase I clinical study of the ^99m^Tc–PD-L1 nanobody was recently completed (35). Notably, Lecocq et al. (41) developed the first anti-LAG-3 nanobodies for SPECT/CT imaging, demonstrating potential applications for detecting tumor-infiltrating immune cells.

### Immune Infiltration Monitoring

In addition to visualizing the tumor's antigen profile, monitoring its immune infiltration regarding density, cell type, and activation levels, can be highly prognostic of a patient's therapeutic response. For monitoring T cell infiltration and activation, an anti-ADP-ribosyltransferase-2 (ART-2) nanobody demonstrated T cell tracking and unexpected therapeutic potential through ART-2 inhibition (42). A PEGylated ^89^Zr-CD8^+^ nanobody could track T cell response to ICP blockade, suggesting the utilization of imaged T cell distributions in predicting ICP therapy response (43). Another study revealed the myeloid compartment's role in PD-1 blockade response using PEGylated ^89^Zr-CD8^+^and ^89^Zr-CD11*b*^+^ nanobodies (44). Jailkhani et al. (45) developed a novel anti-EIIIB nanobody (splice variant of fibronectin) that enhanced detection of tumors, metastasis, and fibroses. The balance between anti-tumor and pro-tumor macrophages is another critical component that dictates the TME; thus, their targeting would be useful in illuminating overall macrophage polarization. The macrophage mannose receptor (MMR) is highly expressed in pro-tumor macrophages (46), and ^99m^Tc-MMR (47), and ^18^F-MMR nanobodies (48) demonstrated specific targeting of MMR^+^ tumor associated macrophages (TAMs), with the ^18^F-MMR possessing a 20x lower kidney retention. Notably, preclinical validation of a ^68^Ga-NOTA-MMR nanobody had no observed toxicity, establishing its qualification for a phase I clinical trial (49). Opposite to MMR, (major histocompatibility complex class II) MHC-II expression is associated with anti-tumor macrophages and indicates effective antigen presentation to CD4^+^ T cells, with ^64^Cu-MHC-II (50), and ^18^F-MHC-II (40) nanobodies demonstrating good T/B ratios. Nanobody-based probes have also been designed to target antigen presenting cells (APCs). De Groeve et al. (51) created ^99m^Tc-labeled nanobodies DC2.1 and DC1.8, mainly targeting myeloid and bone marrow-derived dendritic cells, respectively.

## Nanobodies as a Cancer Therapeutics

### Targeting Tumor Antigens

In 2007, Roovers et al. (52) published the first successful implementation of therapeutic nanobodies for solid tumors *in vivo*. Their anti-EGFR nanobody effectively delayed tumor growth (52), and they later developed a biparatopic version that superiorly reduced EGFR activation, with comparable potency to its mAb counterpart, cetuximab (53). Furthermore, variations have been developed against EGFR's dimer interface (54), EGFR-tyrosine kinase (55), and notably, nanobodies targeting EGFR-ectodomains could overcome the therapeutic resistance associated with mAbs (56). Notably, Rossotti et al. (57) reported DNA immunization-raised EGFR nanobodies with improved functionality compared to protein immunization-raised nanobodies. Nanobodies targeting EGF (58), HER2 (59, 60), CAIX (61), death receptor 5 (DR5) (62, 63), c-Met (64, 65), HGF (66), AgSK1 (67), mesothelin (68), proteasome activator complex PA28 (69), ephrin receptor A4 (EphA4) (70), CEA-cell adhesion molecule-6 (CEACAM6) (71), mitochondrial translation elongation factor (TUFM) (72), protein C receptor (73), Wnt receptors (LRP5/6) (74), and CD33 (75) have also demonstrated delayed tumor growth.

### Nanobody-Based Immune Checkpoint Inhibitors

The inhibition of ICP pathways using mAbs as immune checkpoint inhibitors (ICIs) created a revolutionary breakthrough in the field of cancer therapeutics. Currently, antibodies targeting the molecules PD-1/PD-L1 and CTLA-4 have been FDA approved (76); however, their potency remains inconsistent, with minimal efficacy in most patients. Thus, the structural advantages of nanobodies show promise in enhancing ICIs. Various studies have created nanobody ICIs for PD-L1 (36, 77–81), enhancing anti-tumor efficacy when combined with its mAb counterpart, avelumab *in vivo* (36). Anti-CTLA-4 nanobodies have also demonstrated anti-tumor effects (39, 82); however, Ingram et al. (39) study suggest that an Fc domain may be needed for clinically-relevant potency. Homayouni et al. (83) developed the first nanobody targeting T-cell immunoglobulin and mucin domain 3 (TIM-3), demonstrating anti-proliferative effects *in vitro*. CD47 is another ICI target due to its involvement in both adaptive and innate immunity. However, because CD47 is also highly expressed in red blood cells, their clinical translation is stunted due to the high risks of anemia and hemagglutination (84). Anti-CD47 nanobodies have demonstrated improved therapeutic efficacy and synergistic potential with other ICIs (85, 86); furthermore, the fusion of an anti-CD47 nanobody with an anti-CD20 mAb showed high *in vivo* potency (87).

### Blocking Angiogenesis

**Figure 2:**
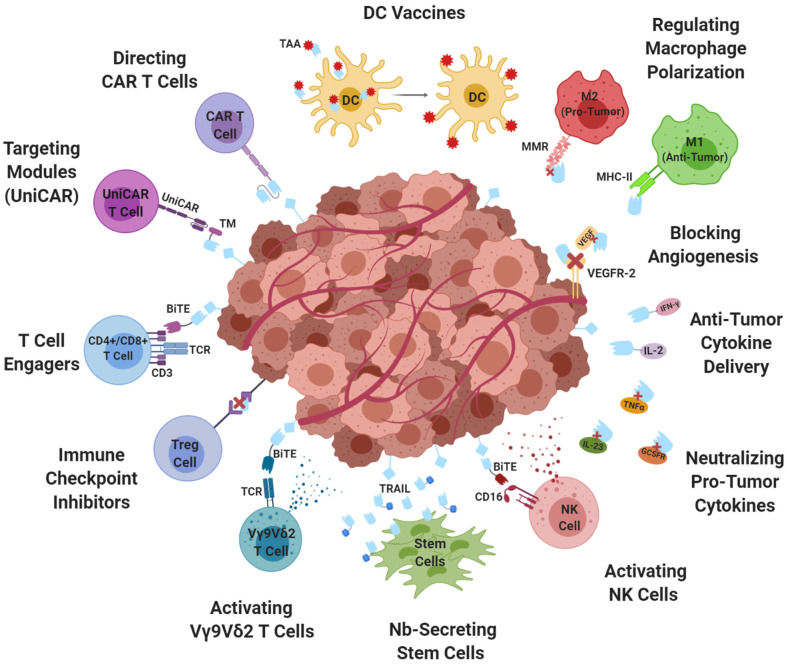
Nanobodies: targeting the tumor microenvironment. The synergistic potential of utilizing nanobodies to enhance tumor therapies targeting the tumor microenvironment. TAA, tumor associated antigen; DC, dendritic cell; MMR, mannose macrophage receptor; MHC-II, major histocompatibility complex-II; VEGF, vascular endothelial growth factor; VEGFR2, vascular endothelial growth factor receptor-2; IFN-γ, interferon- γ; IL-2, Interleukin-2; TNFα, tumor necrosis factor- α; IL-23, Interleukin-23; GCSFR, granulocyte colony-stimulating factor receptor; BiTE, bispecific T cell engager; CD16, cluster of differentiation-16; NK, natural killer; TRAIL, tumor necrosis factor- related apoptosis-inducing ligand; TCR, T-cell receptor; Treg, regulatory T cells; CAR, chimeric antigen receptor; UniCAR, universal CAR; TM, targeting module.

Nanobodies have also demonstrated potential in fighting tumor angiogenesis (Figure 2), a key accelerant of tumor growth and metastasis. The vascular endothelial growth factor (VEGF) and its receptors are well-established stimulants and thus ideal targets for inhibition. Monovalent and bivalent nanobodies blocked VEGF ligand binding (88, 89) while also inhibiting VEGF-activated proliferation *in vivo* (89). Additionally, conjugation to a proline-alanine-serine (PAS) sequence was reported to improve *in vivo* functionality and pharmacokinetics (90). An anti-VEGF receptor-2 (VEGFR2) nanobody demonstrated *in vitro* inhibition of capillary-like formation (91). Furthermore, nanobodies targeting delta-like ligand 4 (DLL4) (92) and CD3 (93) have demonstrated inhibition of neovascularization and tumor proliferation *in vitro* (92) and *in vivo* (93).

## Nanobodies: Synergy With Other Cancer Therapeutics

In addition to intrinsically therapeutic behavior, nanobodies can be utilized to augment the efficacy of other cancer therapies, especially in targeting the TME (Figure 2).

### T Cell Engagers

Antibodies targeting CD3, a receptor found in all T cells, were the first FDA-approved mAbs for clinical use; however, their initial systemic toxicity helped launch the development of bi-specific T-cell engagers (BiTEs). Smaller than mAbs, BiTEs are composed of two scFvs (one activates T cells, the other binds tumor antigens), and nanobody substitution has enabled more compact, enhanced BiTEs. HER2-scFvCD3 (94) and HER2-EGFR (95) BiTEs have been developed that can activate T cell-mediated, targeted tumor lysis both *in vitro* and *in vivo* (94, 95). Li et al. (96) created a BiTE composed of an anti-CEA nanobody and anti-CD3 Fab (“S-Fab”), with significant T-cell mediated cytotoxicity *in vitro* and *in vivo*. The S-Fab was PEGylated to extend its serum half-life and reported uncompromised anti-tumor activity (97). Various advancements have also been made in targeting CD3 (98), and anti-CD3 nanobodies have recently reported a targeted anti-tumor response *in vivo* (93, 99).

Similarly, bispecific light T-cell engagers (LiTEs) targeting EGFR and CD3 have demonstrated T cell-mediated tumor lysis with minimal cytotoxicity (100). The same group subsequently created the “ATTACK,” composed of three anti-EGFR nanobodies and an anti-CD3 scFv, with a 15-fold higher efficacy than their LiTEs (101). The group also developed 4-1BB-agonistic trimerbodies targeting EGFR (102) and CEA (103), with minimal off-tumor cytotoxicity *in vitro* (103) and *in vivo* (102).

### Nanobody-Based CAR-T Cells

The efficacy of chimeric antigen receptor (CAR) T cells has been established in blood-based malignancies; however, their solid tumor implementation has been limited by their inherent immunogenicity and large size of CARs. Various studies have demonstrated the efficacy of utilizing MUC-1 (104), CD7 (105), CD38 (106), VEGFR2 (107), prostate-specific membrane antigen (PSMA) (108, 109), glypican-2 (GPC2) (110), and T cell receptor (TCR)-like nanobody-CARs (111) in various tumor models. Bispecific nanobody-CARs targeting CD20 and HER2 have also been developed; however, experiments have yet to be performed *in vivo* (112). Xie et al. (113) created TME-targeting CAR T cells binding to PD-L1 or EIIIB, with significant tumor reduction *in vivo* and suggested potential in combination therapies. Additionally, anti-PD-L1/CTLA-4 nanobody-secreting CAR T cells have demonstrated enhanced anti-tumor response *in vivo* and indicate synergistic potential (114).

### Targeting Modules (UniCAR)

Studies have also evaluated the integration of nanobodies and the universal CAR (UniCAR) platform. Rather than recognizing tumor antigens, the UniCAR is activated by externally-administered “targeting modules” (TMs), which are UniCAR-activating epitopes linked to an antibody component that recognizes the target antigen, creating a “safety switch” that regulates an otherwise uncontrolled therapy (115). Albert et al. (116) created a nanobody-based TM that effectively retargets UniCAR T cells to induce EGFR^+^ tumor lysis, and they subsequently developed a bivalent version that could target low EGFR^+^ tumors *in vivo* (117).

### γδ T Cell Activators

Gamma-delta (γδ) T cells comprise 0.5–5% of all T-lymphocytes, and the Vγ9Vδ2 T subset has demonstrated therapeutic potential in various tumor models. In the context of nanobodies, a BiTE targeting the EGFR and Vγ9Vδ2 TCR stimulated T-cell mediated cytotoxicity against EGFR^+^ tumor cells *in vivo* (118). Compared to other anti-CD3-BiTEs, this removed the risk of activating pro-tumor cells such as regulatory T cells (Tregs), which heavily predominate the TME.

### Natural Killer Cell Activators

Natural killer (NK) cells possess both cytolytic and immunomodulatory abilities against tumor cells and have demonstrated clinical efficacy in blood-based malignancies. However, like T cell-based therapies, their potency remains stunted in solid tumors, particularly from limited tissue penetration and immunosuppression. To address this, studies have conjugated anti-CD16 nanobodies to nanobodies targeting CEA (119–122), MUC-1 (123), HER2 (124), or to Fabs targeting HER2 (125, 126) or GPC3 (127), with potent tumor growth suppression *in vivo*.

### Dendritic Cell Vaccines

Dendritic cells (DCs) are the most potent of APCs and are promising targets for cancer vaccines, which build the anti-tumor response by prematurely delivering tumor antigen-mAb conjugates to APCs. Utilizing the penetration capacity and structural simplicity of nanobodies, studies have explored the implementation of nanobody-based antigen conjugates to enhance DC-based immunity. Some target DC surface proteins such as CD11b (128, 129), CD36 (128), and MHC-II (128, 130), and others have been designed to block ICPs CTLA-4 (131), and PD-L1 (132) to enhance DC-mediated T cell activation. Kwon et al. (133) developed a novel anti-MHC-II nanobody conjugated to cyclotides (cyclic, plant-derived peptides) that also demonstrated cyclotide-scaffold potential against constrained epitopes. Antigen delivery can also be achieved through viral transduction. Adeno-associated viruses (AAVs), adenoviruses (Ads), and lentiviruses (LVs) have been explored; however, their main limitation is their broad tropism (134). Thus, the surface fusion of nanobodies can increase their target specificity. Nanobody-LVs have shown specific DC transduction (135) *in vitro*, but a subsequent study suggested that broad tropism LVs may be more effective in inducing an anti-tumor response (136). Furthermore, Crowley et al. (137) developed nanobody-conjugated peptide vaccines targeting MHC-II for APC delivery, demonstrating enhanced vaccine-mediated CD8^+^ T cell activation *in vivo*.

### Viral Vectors

Nanobody-AAVs have shown success in targeting antigens such as CD38, ARTC2.2, and P2X7, but further *in vivo* studies are needed (138). Viral vectors have also shown potential for targeting tumor vasculature, and Ahani et al. (139) developed anti-VEGFR2-LVs with comparable targeting to that of VEGF. Additionally, recombinant lambda (λ) bacteriophages have reported significant *in vitro* inhibition of HER2^+^ cell proliferation (140).

### Cytokine Targeting and Delivery

Despite the preclinical success of cytokine-based therapy, clinical studies have been met with subpar efficacy due to their narrow therapeutic window and short half-life. However, the incorporation of nanobodies could impart a new wave of improved cytokine therapies. An anti-PD-L1 nanobody fused to either interleukin-2 (IL-2) or interferon-γ (IFNγ) demonstrated *in vivo* efficacy in treatment-resistant pancreatic tumors (141). Similarly, an anti-CEA nanobody fused to IL-12 demonstrated amplified immune cell proliferation and antitumor activity *in vivo* (142). Furthermore, nanobodies designed to neutralize TNFα (143), IL-23 (144), granulocyte colony-stimulating factor receptor (G-CSF-R) (145), and transforming growth factor beta (TGF-β) (146) have demonstrated success *in vitro* (145), and *in vivo* (143, 144). Nanobodies have also been created to target chemokines, a class of chemotactic cytokines that directly impact tumor proliferation, angiogenesis, and metastasis. Nanobodies blocking protumor chemokines [CXCL10 (147)] or fused to anti-tumor chemokines [CCL21 (148)] have demonstrated functionality in microfluidic devices, but have yet to be tested *in vivo*. Antagonistic nanobodies for chemokine receptors such as ChemR23 (149), US28 (150), CCR7 (151), CXCR4 (152–154), and CXCR7 (154, 155) have also been developed. Smolarek et al. (156) developed the first nanobody inhibiting the Duffy antigen receptor for chemokines (DARC), but it has yet to be applied in tumor models. An anti-L-plastin nanobody was reported to augment T cell proliferation and IL-2 secretion, but this has also not been studied in tumors (157).

### Immunotoxin and Drug Conjugates

Several studies have explored utilizing nanobodies to deliver bacterial toxin pseudomonas exotoxin A (PE). PE and its fragments have been fused to anti-GPC3 (158, 159), anti-GPC2 (110), anti-VEGFR2 (160), anti-CD7 (161, 162), anti-HER2 (163), and anti-CD38 (164) nanobodies, enhancing cytotoxic effects in various tumor models. Notably, Cao et al. (165) developed an enhanced anti-HER2-PE toxin that improved both efficacy and the maximum tolerated dose. β-lactamases from *Enterobacter cloacae* also have established anti-tumor activity, and their enhanced potency after anti-CEA nanobody conjugation reflects potential in improving directed enzyme prodrug therapies (166). Massa et al. (167) conjugated anti-CD20 nanobodies to *Salmonella* bacteria carrying a drug-converting enzyme, demonstrating significant *in vivo* efficacy. L-DOS47 is a recently developed nanobody-urease enzyme conjugate targeting CEACAM6 (168) and is currently in phase I/II clinical trials. A similar anti-VEGFR2 nanobody conjugated to DOS-47 has been developed for angiogenesis inhibition (169). Vlaeminck et al. (170) developed an anti-MMR nanobody fused to an active form of second mitochondria-derived activator of caspase (tSMAC) to target TAMs, reporting upregulated macrophage caspase 3/7 activity *in vitro*. Fusion of anti-EGFR nanobodies have increased therapeutic efficacy of platinum prodrugs (171) and cucurmosin (172), and anti-MHC-II nanobodies fused to the drug DM1 have also exhibited significant targeting and tumor cytotoxicity (173).

### Targeting Moieties for Nanoparticles

Compared to nanobody-drug conjugation, using nanoparticles (NPs) as drug carriers offers benefits such as enhanced protection, bioavailability, and decreased immunogenicity, with enhanced targeting through nanobody conjugation. Wang et al. (174) created quantum-dot-based, anti-EGFR-nanobody micelles carrying aminoflavone, showing enhanced tumor regression *in vivo*. Additionally, anti-EGFR-nanobody micelles carrying doxorubicin (175, 176) and anti-EGFR-nanobody liposomes carrying kinase inhibitors (177) demonstrated enhanced anti-tumor efficacy *in vivo* (176, 177). Interestingly, empty anti-EGFR-nanobody liposomes could also downregulate *in vivo* EGFR expression, an effect that anti-EGFR-scFv liposomes were unable to induce (178). Co-delivery of simvastatin/gefitinib using anti-PD-L1-nanobody liposomes reversed tyrosine kinase inhibitor (TKI) resistance, addressing a major treatment obstacle in non-small-cell lung cancers (NSCLC) (179). Anti-CD8-nanobody-gold NPs carrying a TGF-β inhibitor demonstrated a 40-fold increase in CD8^+^ T cell uptake *in vivo* (180). Polymer-based NPs (181) composed of albumin (182, 183), and polyethyleneimine-PEG (184) demonstrated tumor proliferation inhibition. Notably, anti-HER2, saporin-loaded NPs demonstrated synergistic efficacy when paired with photochemical internalization (PCI) (185). Other explored implementations are nanobody-conjugated extracellular vesicles (186, 187), dendrimers (188), DNA nanoplatforms (189), and nanogels (190), but further studies are needed to characterize these modalities.

### Tumor Penetrating Peptides

Another approach to improving drug uptake is the use of tumor penetrating peptides (TPPs), which can increase vasculature and tissue permeability through activating endocytic pathways (191). Various studies have conjugated nanobodies to TPPs to improve specificity and penetration. Anti-EGFR nanobodies fused to the “iRGD” TPP have demonstrated antitumor activity *in vivo* (192–195), while also showing synergy with chemotherapy drugs (193), T cells (194), silk fibroin nanoparticles (192), and nanoparticles containing gambogic acid (195). Additionally, anti-EGFR nanobodies conjugated to a lactoferrin sequence demonstrated enhanced combinatorial EGFR inhibition (196).

### Nanobody-Secreting Stem Cells

Stem cells have demonstrated great potential in cancer therapeutics due to their inherent tumor tropism and engineered ability to secrete various therapeutic agents, creating a customizable system for targeted tumor delivery. Van de Water et al. (197) developed therapeutic stem cells (SCs) that secreted either anti-EGFR nanobodies (“ENbs”) or proapoptotic ENbs conjugated to TNF-related apoptosis-inducing ligand (TRAIL) for application in GBMs. Notably, the ENb-TRAIL conjugates could significantly reduce tumor growth and invasion *in vivo* across a wide spectrum of GBMs with varying TRAIL resistances (197).

### Nanobodies in α-Particle Radiation Therapy

Targeted radiotherapy delivers a cytotoxic radionuclide-mAb conjugate to the tumor site, and α-particles are commonly used for their high biological efficacy (198). However, their short half-lives are incompatible with the large size, and slow tissue clearance of mAbs; thus, nanobodies hold potential as improved delivery vectors. ^225^Ac-HER2 (199) and ^211^At-HER2 (200) nanobody conjugates enhanced targeting *in vivo* and demonstrate the relevance of further exploring nanobody-delivered α-particle radiation therapy.

### Nanobodies in Photodynamic Therapy

Another avenue of nanobody applications is photodynamic therapy (PDT), which uses a light-activated photosensitizer (PS) to kill tumor cells. mAbs have been used as conjugates to better direct the photosensitizing agent to the tumor site; however, their limitations hinder PDT efficacy and prolong patient photosensitivity (201), which could be circumvented with nanobodies. Anti-HER2 nanobodies conjugated to branched gold NPs could remove HER2^+^ cells upon 5 min of laser treatment (202), and anti-EGFR (203–205), anti-c-Met (205), and anti-U28 (206) nanobody-PS conjugates demonstrated targeted phototoxicity *in vitro* (203, 205, 206), and *in vivo* (203). Additionally, anti-EGFR nanobodies conjugated to a novel Ru^II^ polypyridyl complex reported EGFR-specific targeting (207).

### Intracellular Targeting

Currently, most therapies are designed for extracellular markers, despite the fact that most tumor signaling is controlled intracellularly (208). Various nanobodies, or “intrabodies,” have been created for human papillomavirus (HPV) oncoproteins E6 (209), E7 (210), with E7 intrabody expression in HPV16^+^ cells significantly delaying their growth (210). Steels et al. (211) developed intrabodies targeting tumor suppressor p53's transactivation domain (TAD) and DNA-binding domain (DBD) (212). The p53-DBD nanobodies unexpectedly inhibited p53 transactivation (212), demonstrating that p53 mechanisms are still not fully understood. Additionally, intrabodies developed against B-cell receptor-associated protein 31 (BAP31), have demonstrated caspase-dependent tumor apoptosis *in vivo* (213).

## Delivery of Nanobodies

Most studies have delivered nanobodies intravenously; however, their small size subjects them to rapid renal clearance, increasing the load/frequency needed to achieve clinically relevant efficacy. As it would be both impractical and wasteful to implement such a treatment regimen, a common approach has been to modify nanobodies to extend their serum half-life. PEGylation, fusion to anti-albumin nanobodies or Fc domains, and multimerization have demonstrated longer serum half-lives; however, the trade-offs are lower tumor penetration and additional manufacturing costs. Although not as highly explored, the utilization of nanobody-secreting carriers could circumvent such issues by ensuring both continuous and localized delivery. Notably, “programmable” bacteria that release CD47-targeting nanobodies in the TME increased tumor regression and metastatic inhibition *in vivo* (214). Similarly, Gurbatri et al. (215) engineered a probiotic system that could release anti-PD-L1 and anti-CTLA-4 nanobodies intratumorally, and a single dose reported efficacy comparable to mAbs *in vivo*, a potentiated systemic immune response, and synergistic potential with granulocyte-macrophage CSF (GM-CSF).

For intracellular delivery, nanobodies can also be encoded within viral vectors to produce intracellular-targeting nanobodies inside the tumor, but further *in vivo* studies are needed. Notably, the bacterial type III protein secretion system (T3SS) has been utilized to deliver nanobodies into tumor cells. Essentially a molecular syringe, the nanobodies are injected into the cytoplasm, and anti-EGFP (216), anti-amylase (217), and anti-GFP (217) nanobodies have demonstrated successful delivery *in vitro* (216, 217), and *in vivo* (216). Currently, the unspecific targeting hinders T3SS potential, but this could be addressed through conjugating nanobodies to the bacteria's surface. For imaging applications, most tumors can be visualized through intravenous delivery of nanobody-based probes. The same cannot be said for brain tumors, as the BBB significantly hinders their uptake. However, a recent study found that intra-arterial administration of nanobody imaging probes dramatically enhanced delivery regardless of BBB status (218), suggesting a potential avenue for circumventing BBB limitations.

## Nanobodies in Cancer Clinical Trials

Relative to the numerous avenues of nanobody applications, very few clinical trials have been completed for cancer (Table 3). This could be attributed to their relative infancy as a cancer therapeutic, heightened by the 2012 clinical trial of a tetravalent nanobody targeting DR5, which was terminated due to unanticipated hepatoxicity (NCT01529307). As mentioned previously, the completed phase I trial for the ^68^Ga-HER2 PET/CT nanobody spurred an ongoing phase II trial that will quantify the metastasis in breast carcinoma patients and assess repeatability (NCT03924466). The same group is currently recruiting for a phase II trial assessing ^68^Ga-NOTA-HER2 nanobody uptake in brain metastasis of breast carcinoma patients (NCT03331601), a phase I/II trial for ^68^Ga-NOTA-MMR-VHH2 nanobodies (NCT04168528), and a feasibility trial for anti-idiotypic nanobodies in multiple myeloma patients (NCT03956615). An early phase I trial for evaluating a ^99m^Tc-HER2 nanobody is projected to finish in June 2020 (NCT04040686). Additionally, a completed phase I study assessing the [^**131**^I]-SGMIB-HER2 nanobody demonstrated no adverse effects after one intravenous dose (NCT02683083). The third completed clinical trial is an early phase I study of a ^99m^Tc-PD-L1 nanobody for NSCLC patients (NCT02978196), which successfully visualized tumor uptake 2 h post-injection (35).

**Table 3:** Nanobodies in cancer clinical trials.

**Nanobody**	**Disease**	**Target**	**Clinical trial**	**Phase**	**Status**	**Ref**.
^68^GaNOTA-Anti-HER2 VHH1	Breast carcinoma	HER2		I	Completed	(17)
^68^GaNOTA-Anti-HER2 VHH1	Metastatic breast carcinoma Locally advanced breast cancer	HER2	NCT03924466	II	Recruiting	
^68^GaNOTA-Anti-HER2 VHH1	Breast neoplasm Breast carcinoma Receptor, ErbB-2	HER2	NCT03331601	II	Recruiting	
^99m^Tc-NM-02	Breast cancer	HER2	NCT04040686	I	Recruiting	
^131^I-SGMIB-Anti-HER2 VHH1	Breast cancer Healthy volunteers	HER2	NCT02683083	I	Completed	
^68^GaNOTA-Anti-MMR VHH2	Malignant solid tumor Breast cancer Head and neck cancer Melanoma (Skin)	MMR	NCT04168528	I/IIa	Recruiting	
^99m^Tc-Anti-PD-L1	Non-small cell lung cancer	PD-L1	NCT02978196	I	Completed	(38)
L-DOS47 + Doxorubicin	Pancreas cancer	CEACAM6	NCT04203641	Ib/II	Recruiting	
L-DOS47 + Cisplatin/Vinorelbine	Lung adenocarcinoma		NCT03891173	II	Recruiting	
KN035 + Trastuzumab/Docetaxel	HER2 + Breast cancer	PD-L1	NCT04034823	II	Not yet recruiting	
KN035	Advanced/Metastatic solid tumors	PD-L1	NCT03248843	I	Unknown	
KN035	Solid tumors Hepatocellular carcinoma	PD-L1	NCT03101488	I	Unknown	
KN044	Advanced solid tumors	CTLA-4	NCT04126590	I	Recruiting	
TC-210 T Cells	Mesothelioma Malignant/Pleura/Pleural/Peritoneum Mesothelioma Cholangiocarcinoma Recurrent cholangiocarcinoma Ovarian cancer Non-small cell lung cancer	Mesothelin	NCT03907852	I/II	Recruiting	
CD19/CD20 bispecific CAR T cells	Refractory/Relapsed B-cell lymphoma stage	CD19/CD20	NCT03881761	I	Recruiting	
BCMA CAR T cells	Refractory/Relapsed myeloma	BCMA	NCT03664661	I	Recruiting	
Anti-idiotypic	Multiple myeloma	Paraproteins	NCT03956615	N/A (Feasibility)	Recruiting	
TAS266	Advanced solid tumors	DR5	NCT01529307	I	Terminated	(12)

*HER2, human epidermal growth factor receptor-2; MMR, macrophage mannose receptor; PD-L1, programmed death ligand 1; CTLA-4, cytotoxic T lymphocyte antigen-4; CEACAM6, carcinoembryonic antigen cell adhesion molecule-6; CD19/CD20, cluster of differentiation 19/20; BCMA, B-cell maturation antigen; DR5, death receptor-5*.

As for therapeutics, phase Ib/II and phase II trials are currently evaluating the safety and tolerability of L-DOS47 in combination with doxorubicin (NCT04203641) or vinorelbine/cisplatin (NCT03891173), respectively. Furthermore, trials testing the safety and tolerability of PD-L1 (NCT04034823, NCT03248843, NCT03101488) and CTLA-4 (NCT04126590) inhibitors are recruiting for breast and metastatic tumor patients. Nanobody-based immune cell therapies also have clinical trials in the recruiting phase. A phase I/II trial will assess the overall response rate to T cells expressing anti-mesothelin nanobodies fused to the endogenous TCR (NCT03907852). CD19/20 (NCT03881761) and B-cell maturation antigen (BCMA) CAR T cells (NCT03664661) will also be assessed in patients with refractory/relapsed B cell lymphoma.

## Perspectives

Nanobodies uniquely possess the combined therapeutic advantages of mAbs and the targeting potential of nanoscale delivery. Their compact size enables enhanced tumor penetration and access to hidden and/or intracellular epitopes, while also granting high modularity for creating more complex nanobody-based constructs. Their robustness and manufacturing ease are favorable for large-scale production, and their superior paratope diversity allows an extensive arsenal for tumor antigen targeting. Although nanobodies could be portrayed as a superior version of mAbs, it is important to consider the implications of their differences. Nanobodies are subject to rapid renal clearance, and although this is desirable for imaging purposes, it limits their therapeutic lifetime and lowers the threshold for inducing renal toxicity (219), further limited if conjugated to cytotoxic loads. However, this could be combatted through infusing gelofusine or lysine (27), inserting charged residues in the nanobodies, and the aforementioned methods of extending serum half-life. Additionally, unlike mAbs, nanobodies lack an Fc region, and thus cannot directly initiate an Fc-mediated immune response.

As nanobodies are not naturally produced in humans, their therapeutic implementation brings into question their overall safety (Table 4). Nanobody sequence studies have revealed high similarity with human VH domains (220), and combined with their size, structure, and low agglutination, nanobodies possess low immunogenicity and are appropriate for human administration. Nonetheless, immunogenicity could be further minimized through “humanization,” which is generally accomplished through replacing various surface regions with human sequences. However, such replacements may compromise functionality, and more concerningly, humanization may decrease solubility, negating any immunogenicity-lowering effects (221). Currently, conflicting clinical results make it difficult to establish an immunogenic profile (222, 223), which may best be resolved through the completion of additional clinical trials.

**Table 4:** Obstacles to clinical translation.

**Consideration**	**Reason**	**Potential solution**
Clinical trial attrition	Failure rates of therapeutic candidates generally increase with each trial phase due to poor translation from preclinical models.	→ Reduce and refine animal-based models → Enhance *in vitro* and *in silico* methods
Administration/Dosing	The short serum half-life of unmodified Nbs requires frequent and concentrated IV delivery for therapeutic applications, which increases the risk of renal toxicity.	→ Extend serum half-life through albumin-tagging, Fc-domain fusion, PEGylation, multimerization → Alternative delivery methods: SCs, viral vectors, programmable bacteria, intracellular delivery, intra-arterial delivery (for BBB)
Immunogenicity	As Nbs come from camelids, they possess a low risk of triggering an immune resposne.	→ Humanization of Nbs → Developing different idiotypes for a specific Nb → Developing “camelized,” fully human HcAbs (substituted hydrophilic residues into hydrophobic regions)
Functionalization	Modification of Nbs to conjugate other molecules or build more complex constructs might compromise their original functionality.	→ Site-selective Nb functionalization
On-Target/On-Tumor cytotoxicity	Excessive targeting could cause adverse effects such as cytokine release syndrome and tumor lysis syndrome.	→ “Safety-switch”/Suicide gene therapy → Separating out initial dosing regimen
On-Target/Off-Tumor cytotoxicity	Tumor antigen could also be expressed on non-malignant cells and cause damage to healthy tissue.	→ “Safety-switch”/Suicide gene therapy → Bispecific activation → Improve imaging of patient's tumor antigen profile to determine toxicity threshold
Reaching clinical-grade efficacy	Success in preclinical models is not necessarily indicative of therapeutic efficacy in human patients	→ Improve Nb affinity maturation → Improve Nb orientation → Enhance ADCC
Quality control	Ensure that Nbs are homogenous to avoid variability in functionality and risk adverse effects	→ Good manufacturing practices for microbial-based Nb production

*Nbs, nanobodies; IV, intravenous; SCs, stem cells; BBB, blood brain barrier; Fc, fragment crystallizable; HcAb, heavy-chain antibodies; ADCC, antibody-dependent cellular cytotoxicity*.

Nanobody are versatile in that their applications extend across the full timeline of a cancer patient's treatment. Using nanobody-based imaging probes has shown improved visualization compared to traditional mAb-based probes. On their own, nanobodies can be utilized as targeted antagonists, ICIs, angiogenesis inhibitors, and as cytokine neutralizers or stimulants. Their synergy with existing cancer therapeutics is reflective of their promising potential to elevate cancer treatments well outside of their origins in antibody-based applications. Nanobodies can be conjugated to drugs, cytokines, NPs, TPPs, photosensitizers, and α-particles for enhanced delivery. Furthermore, they can augment immune cell-based therapies, improve viral vector delivery, and be secreted by engineered stem cells and bacteria. In light of these various applications, their greatest potential may be found in intracellular targeting. As evidenced by existing preclinical studies, the targeting of critical intracellular tumor antigens may be the next pivotal step to revolutionizing a new wave of cancer therapeutics.

## Author Contributions

EY and KS: literature review and interpretation, manuscript writing, and final approval of manuscript. KS: conception and design. Both authors contributed to the article and approved the submitted version.

## Conflict of Interest

KS owns equity in and is a member of the Board of Directors of AMASA Therapeutics, a company developing stem cell-based therapies for cancer. KS's interests were reviewed and are managed by Brigham and Women's Hospital and Partners HealthCare in accordance with their conflict of interest policies. The remaining author declares that the research was conducted in the absence of any commercial or financial relationships that could be construed as a potential conflict of interest.
